# Patient positioning in minimally invasive gynecologic surgery: strategies to prevent injuries and improve outcomes

**DOI:** 10.61622/rbgo/2024rbgo46

**Published:** 2024-05-27

**Authors:** Agnaldo Lopes da Silva, Matheus Eduardo Soares Pinhati, Gabriel Lage Neves, Eduarda Naves Gonçalves de Almeida, Teresa Lamaita Lopes, Rívia Mara Lamaita, Eduardo Batista Cândido

**Affiliations:** 1 Universidade Federal de Minas Gerais Department of Gynecology and Obstetrics Belo Horizonte MG Brazil Department of Gynecology and Obstetrics, Universidade Federal de Minas Gerais, Belo Horizonte, MG, Brazil.; 2 Universidade Federal de Minas Gerais School of Medicine Belo Horizonte MG Brazil School of Medicine, Universidade Federal de Minas Gerais, Belo Horizonte, MG, Brazil.; 3 Faculdade Ciências Médicas de Minas Gerais Belo Horizonte MG Brazil Faculdade Ciências Médicas de Minas Gerais, Belo Horizonte, MG, Brazil.

**Keywords:** Minimally invasive surgical procedures, Gynecologic surgical procedures, Patient positioning, Treatment outcome, Intraoperative complications, Nerve injury, Patient safety, Compartment syndrome, Pressure ulcer, Risk factors

## Abstract

Effective patient positioning is a critical factor influencing surgical outcomes, mainly in minimally invasive gynecologic surgery (MIGS) where precise positioning facilitates optimal access to the surgical field. This paper provides a comprehensive exploration of the significance of strategic patient placement in MIGS, emphasizing its role in preventing intraoperative injuries and enhancing overall surgical success. The manuscript addresses potential complications arising from suboptimal positioning and highlights the essential key points for appropriate patient positioning during MIGS, encompassing what the surgical team should or shouldn't do. In this perspective, the risk factors associated with nerve injuries, sliding, compartment syndrome, and pressure ulcers are outlined to guide clinical practice. Overall, this paper underscores the critical role of precise patient positioning in achieving successful MIGS procedures and highlights key principles for the gynecological team to ensure optimal patient outcomes.

## Introduction

Effective patient positioning stands as a pivotal determinant influencing the occurrence of surgical complications and the achievement of favorable patient outcomes. Within the realm of minimally invasive gynecologic surgery (MIGS), the significance of precise patient positioning cannot be overstated, as proper patient alignment is instrumental in harnessing the potential benefits associated with MIGS. The advantages of proper patient positioning in MIGS include better visual and technical access to the surgical site, diminished risk of intraoperative injuries, elevated levels of patient comfort and lower rates of unnecessary exposure.^(1,2)^

The genesis of appropriate patient positioning may be traced back to seminal instances in the history of gynecologic surgery. Early laparoscopic hysterectomies, which were pioneered by Reich et al. ^(3)^ in 1989, and the inaugural series of robotic-assisted hysterectomies documented by Diaz-Arrastia et al. ^(4)^ in 2002, underscored the employment of lithotomy and Trendelenburg positioning to facilitate endoscopic access to the intricate pelvic region.

Surgical positioning in MIGS mandates a standardized and meticulous approach, requiring a multidisciplinary effort by gynecologists, anesthetists and perioperative nurses.^(5)^

In this manuscript, we undertake a thorough examination of patient positioning in MIGS, highlighting the critical role of strategic placement in minimizing intraoperative injuries and enhancing surgical outcomes. We elucidate the risk factors and primary complications arising from suboptimal positioning, encompassing complications associated with Trendelenburg positioning, neuropathies, compartment syndrome, and skin ulcers. Moreover, we present the key points for appropriately positioning a patient during MIGS. This revision aims to succinctly articulate the critical role of patient positioning in MIGS, emphasizing the importance of preoperative preparation and the value of time invested in patient positioning to improve surgical outcomes.

## Patient positioning

### Head and neck

**Figure 1:**
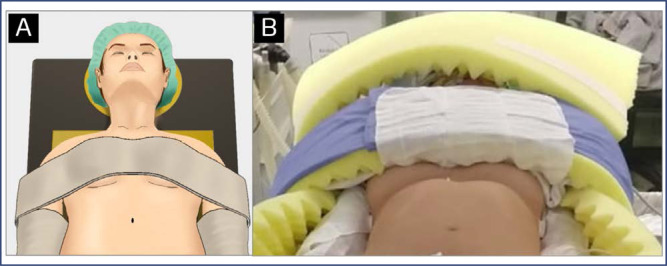
Head should be positioned in the midline of the operating table (A). Example of proper protection of face with a small piece of egg foam mattress (B)

Source: A: Illustration by Matheus Eduardo Soares Pinhati (authorized publishing). B: Photografic record of the authors.

The head should be stabilized in a central position with appropriate material that prevents lateral flexion or hyperextension during surgery. The cervical spine should be stabilized in a neutral position without any pressure points on the back of the head.^(5–7)^ There are no standard recommendations for face and eye protection. The risk of facial trauma during robotic surgery is higher, especially when the trocars are positioned above the umbilical scar, making it possible for an arm to reach the facial region. The face can be protected, for instance, with a small piece of egg foam mattress or an equivalent accessory (Figure 1).^(1,8)^

### Upper limb

For minimally invasive surgeries, the arms must be placed along the patient's body with the palms facing the side of the thigh and the elbows, wrists and hands protected with padded material. The area between the armpit and the forearm should also be protected with padding to reduce pressure on the radial and ulnar nerves. The forearm might remain in a neutral position (with thumbs up). The elbow should not be in direct and prolonged contact with the operating table, mainly in its posteromedial region, where the ulnar nerve passes. Hands should be protected, avoiding contact with metal surfaces and compression. All peripheral venous accesses and monitoring arrangements should be protected to reduce pressure on the patient's skin. The arm is secured by wrapping the sheet previously positioned under the anti-slip mattress over the arm and placing it under the side of the patient's back. Compresses and adhesive tapes can be used to optimize arm fixation. The shoulders should remain in a neutral position, so that the upper limb should be positioned with the main aim of avoiding shoulder depression. To ensure the stability of the shoulders in this position, non-slip padding may be used. If shoulder supports are used, they should be placed directly over the acromioclavicular joint.^(1,2,5,7,9)^

### Pelvis and lower limb

**Figure 2:**
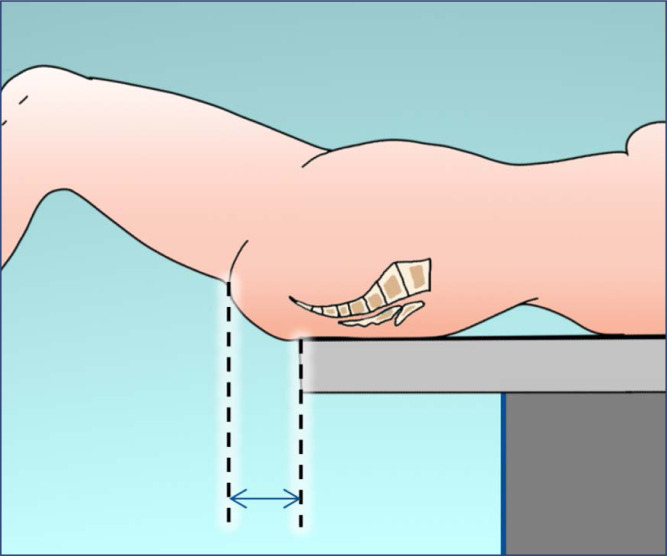
The buttocks should be positioned slightly off the edge of the operating table

Source: Illustration by Matheus Eduardo Soares Pinhati (authorized publishing).

**Figure 3:**
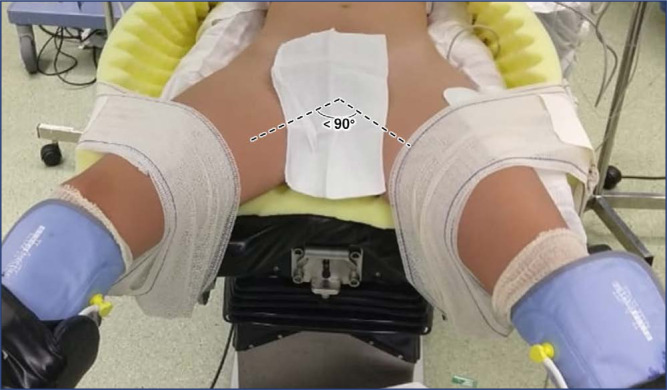
Thigh abduction should not exceed 90 degrees

Source: Photografic record of the authors.

In lithotomy position, the legs should remain flexed and the buttocks should be placed slightly above the edge of the operating table, with the sacrum properly supported by soft materials. If a uterine manipulator is required, positioning the buttocks about five to ten centimeters off the lower edge of the operating table may increase the range of motion of the manipulator (Figure 2). The position may be referred to as exaggerated lithotomy, high lithotomy, standard lithotomy, or low lithotomy, depending on the degree of hip flexion. The ideal position for MIGS is low lithotomy with a thigh-trunk angle of 170° and with the knees above the abdominal plane. This positioning allows for proper docking with less risk of collision between the robotic arms and the patient's legs. Padded boot stirrups are recommended (e.g., Allen stirrups, Yellowfin stirrups) as they provide full ankle and foot support with minimal pressure on the head of the fibula. The padding of the lower limb over the bony prominences prevents compression of the legs against the supports. Candy cane stirrups are unsuitable for MIGS because they require complete immobility during the procedure. The thighs should be abducted, the angle between them should not exceed 90° (Figure 3), and lateral rotation of the thigh should be minimal. Any thigh abduction greater than 60° must be accompanied by hip flexion. The knee should be flexed at an angle between 90° and 120°. Both stirrups should be raised to the same height at the same time to avoid possible sprains and injuries in the lumbar region. At the end of the operation, reassemble the leg section of the table and bring the legs into a horizontal position.^(1,5,7–11)^

### Trendelenburg positioning and related complications

**Figure 4:**
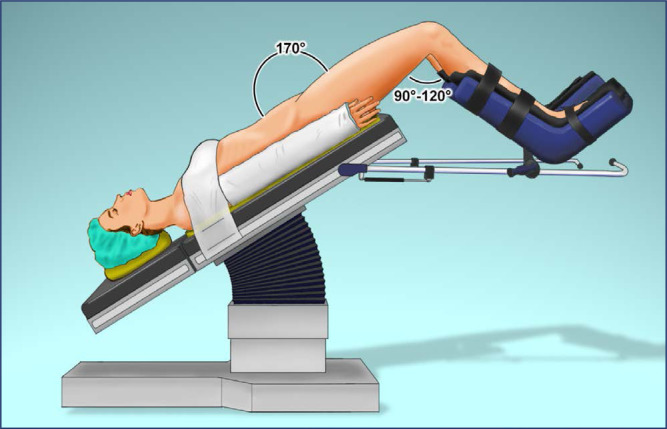
Proper positioning in steep Trendelenburg, with a friction pad under patient, chest straps, thigh-trunk angle of 170° and knees flexion of 90°-120°

Source: Illustration by Matheus Eduardo Soares Pinhati (authorized publishing).

In the Trendelenburg position, the operating table is tilted so that the head and trunk are lower than the lower extremities, optimizing exposure and access to the pelvis (Figure 4). The combination of the Trendelenburg position and lithotomy of the lower limbs is intended to simultaneously expose and provide access to the abdomen and perineum, allowing, for example, the use of the uterine manipulator by the assistant surgeon.^(10,12)^ The appropriate tilt should be able to mobilize the small intestine and colon toward the upper abdomen, permitting visualization of the promontory and the right ureter crossing the bifurcation of the common iliac artery. There is no consensus on the angles that define Trendelenburg, with variations in the literature ranging from 20° to 45°. The average angle used in robotic gynecologic surgery (benign and malignant) is 28° (95% CI, 26.9-29.1), with angles of less than 30° allowing more than the most of procedures to be performed (95% CI, 50%-70%). Angles of less than 20° appear to be sufficient for gynecologic surgery for benign indications (mean, 16.4°; 95% CI, 14.4-18.3). Although, more severe Trendelenburg inclinations are required for lymphadenectomy in oncologic surgery.^(10,13,14)^

The steep Trendelenburg position can have significant effects on the pulmonary and cardiovascular systems. Furthermore, there is a risk of slippage on the operating table and brachial plexus injury. A survey of American Society of Anesthesiologists (ASA) members showed that 21.7% of consultants had experienced a complication related to Trendelenburg positioning during robotic surgery.^(15)^

Some of the complications arising from the Trendelenburg position include cardiac arrhythmias and increased intracranial and intraocular pressure. Neurological and cognitive complications, partial or total loss of vision and retinal dislocation are rare. Patients with a history of comorbidities in any of these systems are likely to be affected. It is therefore advisable that patients who have underlying lung conditions such as sleep apnea, chronic obstructive pulmonary disease, restrictive lung disease, heart disease, cerebrovascular disease, and eye conditions (such as glaucoma) should be properly assessed by the respective specialist before undergoing surgery. Other complications involving the head and face include otorrhagia and alopecia, also hypertension appears to be a possible risk factor.^(8,16)^

Another important complication that can arise from the Trendelenburg position is the risk of slippage in the operating table. The main risk factor for that complication is a BMI > 30 kg/m^2^. Several devices can be used to reduce the risk of patient sliding, such as egg crate, memory foam, gel pad, shoulder straps, chest straps, and bean bag. Das et al.^(2)^ evaluated the mean cephalad patient slide distance using a selection of these devices in a 30 to 35^o^ deep Trendelenburg position, with distances ranging from 1.07 ± 1.93 cm to 4.5 ± 4.0 cm. Best evidence recommendations cannot be made for a specific device. While cephalad slide is generally uncommon among the wide variety of devices available, it is important to consider the pricing of these devices.^(2,17)^

The employment of shoulder supports as a countermeasure to cephalad displacement during the deep Trendelenburg position remains contentious. An analytical survey identified that, out of 24 case studies detailing brachial plexus injuries subsequent to laparoscopic procedures, 63% were linked with pelvic surgeries where the Trendelenburg posture was implemented.^(6)^ Placing the supports more medially in relation to the acromioclavicular joint may compress the proximal roots of the brachial plexus, while placing the supports more laterally can stretch the brachial plexus. However, the use of supports was not associated with an increased incidence of brachial plexus injuries in a systematic review.^(2)^

## Complications

### Nerve injury

The incidence of nerve injuries during minimally invasive surgery in gynecology is 0.16%. The main affected nerves, the causes, symptoms, and specific prevention strategies are summarized in chart 1. In general, a good rule is to position and pad exposed peripheral nerves in order to prevent their stretch beyond normally tolerated limits while the patient is awake; avoid their direct compression, if possible; and distribute any compressive forces that must be placed on the nerves over an area which is possible.^(18)^ Some potential risk factors for nerve injuries include duration of lithotomy position > 2h, previous abdominal surgery, BMI < 20 or > 30 kg/m^2^, age > 70 years, peripheral arterial disease and diabetes.^(2,5,17–19)^

**Chart 1:** Nerve injuries

Nerve injury	Causes	Symptoms	Recommendations for prevention
Ulnar nerve	Direct pressure in the elbow Pronation of the arm on the arm braces Surgeon leaning against the patient arm	Paresthesia of the fourth and fifth fingers and on the ulnar side of the hand. "Ulnar claw"	Place the forearm in a neutral position. Avoid flexion of the elbow to decrease the risk of ulnar neuropathy.
Brachial plexus (not related with Trendelenburg)	Dropping away of the shoulder girdle in an anesthetized, relaxed patient. Hyperextension combined with rotation at the cervical spine.	Motor and sensory deficits in the shoulder, upper and lower arms, and hands.	Avoid excessive lateral rotation of the head. Limit abduction of the arm to < 90 degrees. Keep the chest roll out of the axilla to avoid neurovascular compression
Sciatic and peroneal nerves	Compression on the peroneal nerve secondary to placement of patients in a lithotomy position Direct pressure injury caused by unpadded contact with the leg holder. Combination of hip flexion and knee extension.	Sensory deficits in the lateral lower leg and at the arch of the foot. Limited dorsiflexion of the foot (foot drop – steppage gait). Weakness of the dorsiflexor muscles of the foot.	Minimize the time in the lithotomy position. Use two assistants to coordinate simultaneous movement of both legs to and from the lithotomy position. Avoid excessive flexion of the hips, extension of the knees, or torsion of the lumbar spine. Avoid excessive pressure on the peroneal nerve at the fibular head.
Femoral nerve	Inadequate hip abduction or flexion.	Deficits in hip flexion and knee extension together with a diminished patellar reflex. Quadriceps femoral paresis.	Avoid extension or flexion of the hip.
Obturator nerve	Abduction of the thigh with an angle of between 30–45° in the hip joint (angle between the thighs 60-90°).	Loss of cutaneous sensation over the medial thigh, with or without weakness of hip adduction	Maximum angle of abduction should not exceed 90°. Abduction of the lower extremity must be accompanied by flexion of the hip joint.

Source: Elaborated by the authors.

### Compartment syndrome

Compartment syndrome occurs when the pressure in any fascial compartment exceeds the arterial perfusion pressure, leading to ischemia and tissue necrosis. Patients undergoing prolonged pelvic surgery can develop acute compartment syndrome of the lower limbs even in the absence of direct trauma to the legs or pre-existing vascular disease (Well Leg Syndrome). Lithotomy position is associated with acute compartment syndrome, with an incidence of 0.028% in gynecological surgeries in general, and up to 0.38% in surgeries with duration of over 180 minutes.^(20,21)^ The calf is the site of the lower extremity most frequently affected, and intracompartmental pressure is influenced by leg supports, especially boot stirrups.^(22,23)^ Leg elevation with Trendelenburg or high lithotomy are also causes of increased intracompartmental pressure.^(24–26)^ Some risk factors of the compartment syndrome include duration of lithotomy position > 2 h, operative time, intraoperative hypotension, BMI > 30 kg/m^2^, peripheral arterial disease and diabetes.

The diagnosis of compartment syndrome is based on clinical findings. The primary symptom is severe post-surgical pain in the legs. Other possible signs include pain when the affected compartment is stretched, numbness or tingling, skin paleness, weakness, and a lack of pulse in the lower limb.^(27)^ The sensitivity of individual findings is low (13%-19%), however, the probability of compartment syndrome increases to 93% when three symptoms are present.^(28)^ There are no evidence-based recommendations on the prevention of compartment syndrome. The use of pulse oximeters to monitor the extremities does not offer benefits such as arterial perfusion and oxygen, as well as intraoperative monitoring of intracompartmental pressure.^(5,20)^ Continuous monitoring of compartment pressure in postoperative patients is not indicated. Despite the lack of evidence, it is recommended to consider resting legs for 10 minutes every 2 hours of surgery.^(29)^

### Pressure ulcers

Pressure ulcers are caused by tissue ischemia resulting from external compression. Bony prominences such as the sacral, ischial and heel regions are common sites for these lesions. The lesion may become visible a few days after the damage has been occurred. Some potential factors for pressure ulcers during the perioperative period include operative time, age > 71 years, dehydration, hypotension, nerve blocks, malnutrition, immunosuppression, peripheral arterial disease, diabetes and patient positioning.^(5,30)^

A prospective study not only found that sacral and ischial pressure ulcers were very common (43% and 15% respectively), but also that the second most common location was the heel (19%). Therefore, to avoid pressure ulcers, it is important to ensure that the weight of the legs is distributed over the entire calf area, avoiding pressure on the Achilles heel. To minimize potential harm, a review by Cochrane analyzed studies examining the efficacy of pressure-relieving mattresses and overlays, also found that their use on the operating table, particularly during lengthy surgeries, decreases the occurrence of pressure ulcers after the procedure. The efficacy was strengthened by a meta-analysis that confirmed the preventative impact of pressure mattresses versus standard foam mattresses.^(5,30,31)^

### Final remarks

The current literature on patient positioning within the scope of MIGS is considerably limited. The extant studies offer limited evidence, and prevailing epidemiological data on postoperative complications largely hinge on dated studies or expert consensus. Position-related injuries in gynecologic surgeries can be severe, potentially leading to serious patient harm. These injuries have the potential to result in significant patient harm and frequently precipitate malpractice litigation, necessitating exhaustive medical review. Positioning-induced complications in MIGS, such as nerve injuries, compartment syndrome, and pressure ulcers, require specialized management approaches. Significant risk factors, including age, obesity, diabetes, and vascular disease, have been linked to an increased probability of these adverse events. Specifically, a body mass index (BMI) over 30 kg/m^2^ can heighten the risk of movement in the Trendelenburg position, and procedures exceeding four hours are more likely to result in positioning-related injuries. Recognizing these factors during preoperative planning and striving to minimize the duration of surgery are critical. Chart 2 delineates the key elements on the critical aspects of patient positioning, from the arrangement of the head and limbs to the application of the Trendelenburg position. Compliance with these recommendations is essential to the success of MIGS, providing healthcare professionals with a definitive guide to ensure precise patient positioning. These practices are instrumental in reducing intraoperative complications and improving surgical success rates, establishing a strategic template for optimal patient placement in MIGS.

**Chart 2:** Key points for optimal patient positioning in minimally invasive gynecological surgery

DOs
Position the head in the midline of the operating table and do not flex it laterally Protect the face and the eyes Use non-slipping friction materials under the patient and chest straps to reduce the risk of sliding Position the arms tucked at sides in a neutral position Keep the shoulders in a neutral position that prevents its depression and posterior displacement If shoulder braces are used, place them at the level of the acromioclavicular joints Position the legs in padded boots stirrups Keep the buttocks at the edge of the table Keep a thigh-trunk angle of 170° Flex the knees at an angle between 90° and 120° Abduct the thighs at angle that should not exceed 90° Keep a minimal external rotation of hip Keep a minimal lateral rotation of thigh. Recheck the positions of the hands, legs and feet after the operative table is brought to Trendelenburg. Consider any physical abnormalities when positioning the patient After surgery, reassemble the leg section of the table and bring the legs into a horizontal position
DON'Ts
Underestimate the importance of patient positioning in MIGS Distribute the weight of the legs unevenly over the calf area Use shoulder braces without using non-slipping friction materials under the patient Keep the boot stirrups raised at different heights Let any parts of the body in direct contact with the metal surfaces of the operating table Let any direct pressure be applied on the Achilles heel, on the elbow and on the fibula neck Let any parts of the body hanging over the edge of the operating table Let the sacrum extend beyond the edge of the operating table Keep the patient more than 4 hours in Trendelenburg position, if possible

Source: Elaborated by the authors.

## Conclusion

In essence, the significance of patient positioning in minimally invasive gynecologic surgery is paramount. It is a critical component in reducing surgical injuries and enhancing patient outcomes. This process demands a coordinated and integrated effort from the entire surgical team to customize positioning strategies to the particulars of each procedure, taking into consideration the unique attributes and clinical history of each patient. An operating room that is well-equipped and a commitment to diligent patient positioning lay the groundwork for minimizing the occurrence of minimally invasive gynecologic surgery related complications. Indeed, the time spent before surgery in ensuring proper positioning can make a substantial difference in surgical outcomes.

## References

[1] Takmaz O, Asoglu MR, Gungor M (2018). Patient positioning for robot-assisted laparoscopic benign gynecologic surgery: a review. Eur J Obstet Gynecol Reprod Biol.

[2] Das D, Propst K, Wechter ME, Kho RM (2019). Evaluation of positioning devices for optimization of outcomes in laparoscopic and robotic-assisted gynecologic surgery. J Minim Invasive Gynecol.

[3] Reich H, DeCaprio J, McGlynn F (1989). Laparoscopic hysterectomy. J Gynecol Surg.

[4] Diaz-Arrastia C, Jurnalov C, Gomez G, Townsend C (2002). Laparoscopic hysterectomy using a computer-enhanced surgical robot. Surg Endosc.

[5] Fleisch MC, Bader W, Balzer K, Bennefeld L, Boeing C, Bremerich D (2021). The prevention of positioning injuries during gynecologic surgery. Guideline of the DGGG, OEGGG and SGGG (S2k Level, AWMF Registry Number 015/077, October 2020). Geburtshilfe Frauenheilkd.

[6] Shveiky D, Aseff JN, Iglesia CB (2010). Brachial plexus injury after laparoscopic and robotic surgery. J Minim Invasive Gynecol.

[7] Abdalmageed OS, Bedaiwy MA, Falcone T (2017). Nerve injuries in gynecologic laparoscopy. J Minim Invasive Gynecol.

[8] Lim PC, Kang E (2017). How to prepare the patient for robotic surgery: before and during the operation. Best Pract Res Clin Obstet Gynaecol.

[9] Arnold A, Abbott J (2014). Complications of laparoscopic surgery. Obstet Gynaecol Reprod Med.

[10] Working Group of ESGE (2019). Surgical steps of total laparoscopic hysterectomy: Part 1: benign disease by the European Society for Gynaecological Endoscopy (ESGE)1. Facts Views Vis Obgyn.

[11] Agostini J, Goasguen N, Mosnier H (2010). Patient positioning in laparoscopic surgery: tricks and tips. J Visc Surg.

[12] Lloyd-Davies OV (1939). Lithotomy-Trendelenburg position for resection of rectum and lower pelvic colon. Lancet.

[13] Gould C, Cull T, Wu YX, Osmundsen B (2012). Blinded measure of Trendelenburg angle in pelvic robotic surgery. J Minim Invasive Gynecol.

[14] Ghomi A, Kramer C, Askari R, Chavan NR, Einarsson JI (2012). Trendelenburg position in gynecologic robotic-assisted surgery. J Minim Invasive Gynecol.

[15] Souki FG, Rodriguez-Blanco YF, Polu SR, Eber S, Candiotti KA (2018). Survey of anesthesiologists’ practices related to steep Trendelenburg positioning in the USA. BMC Anesthesiol.

[16] Arvizo C, Mehta ST, Yunker A (2018). Adverse events related to Trendelenburg position during laparoscopic surgery: recommendations and review of the literature. Curr Opin Obstet Gynecol.

[17] Steck-Bayat KP, Henderson S, Aguirre AG, Smith RB, Mahnert NM, Gerkin RD (2020). Prospective randomized controlled trial comparing cephalad migration in robotic gynecologic surgery using egg-crate foam versus the Pink Pad®. J Robot Surg.

[18] Warner MA (1998). Perioperative neuropathies. Mayo Clin Proc.

[19] Bauer EC, Koch N, Janni W, Bender HG, Fleisch MC (2014). Compartment syndrome after gynecologic operations: evidence from case reports and reviews. Eur J Obstet Gynecol Reprod Biol.

[20] Gill M, Fligelstone L, Keating J, Jayne DG, Renton S, Shearman CP (2019). Avoiding, diagnosing and treating well leg compartment syndrome after pelvic surgery. Br J Surg.

[21] Bauer EC, Koch N, Erichsen CJ, Juettner T, Rein D, Janni W (2014). Survey of compartment syndrome of the lower extremity after gynecological operations. Langenbecks Arch Surg.

[22] Halliwill JR, Hewitt SA, Joyner MJ, Warner MA (1998). Effect of various lithotomy positions on lower-extremity blood pressure. Anesthesiology.

[23] Meyer RS, White KK, Smith JM, Groppo ER, Mubarak SJ, Hargens AR (2002). Intramuscular and blood pressures in legs positioned in the hemilithotomy position: clarification of risk factors for well-leg compartment syndrome. J Bone Joint Surg Am.

[24] Martin JT (1992). Compartment syndromes: concepts and perspectives for the anesthesiologist. Anesth Analg.

[25] Peters P, Baker SR, Leopold PW, Taub NA, Burnand KG (1994). Compartment syndrome following prolonged pelvic surgery. Br J Surg.

[26] Horgan AF, Geddes S, Finlay IG (1999). Lloyd-Davies position with Trendelenburg—a disaster waiting to happen?. Dis Colon Rectum.

[27] Tiwari A, Haq AI, Myint F, Hamilton G (2002). Acute compartment syndromes. Br J Surg.

[28] Ulmer T (2002). The clinical diagnosis of compartment syndrome of the lower leg: are clinical findings predictive of the disorder?. J Orthop Trauma.

[29] Harris IA, Kadir A, Donald G (2006). Continuous compartment pressure monitoring for tibia fractures: does it influence outcome?. J Trauma.

[30] Boyko TV, Longaker MT, Yang GP (2018). Review of the current management of pressure ulcers. Adv Wound Care (New Rochelle).

[31] Shi C, Dumville JC, Cullum N, Rhodes S, Jammali-Blasi A, McInnes E (2021). Alternating pressure (active) air surfaces for preventing pressure ulcers. Cochrane Database Syst Rev.

